# Inhibition of *E. coli* Growth by Nanodiamond and Graphene Oxide Enhanced by Luria-Bertani Medium

**DOI:** 10.3390/nano8030140

**Published:** 2018-03-01

**Authors:** Jaroslav Jira, Bohuslav Rezek, Vitezslav Kriha, Anna Artemenko, Iva Matolínová, Viera Skakalova, Pavla Stenclova, Alexander Kromka

**Affiliations:** 1 Institute of Physics, Academy of Sciences of the Czech Republic, Cukrovarnická 10, 162 00 Prague 6, Czech Republic; artemenko@fzu.cz (A.A.); artemenko@fzu.cz (P.S.); kromka@fzu.cz (A.K.); 2 Faculty of Electrical Engineering, Czech Technical University, Technická 2, 166 27 Prague 6, Czech Republic; rezekboh@fel.cvut.cz (B.R.); kriha@fel.cvut.cz (V.K.); 3 Faculty of Mathematics and Physics, Charles University, V Holešovičkách 2, 181 00 Prague 8, Czech Republic; imatol@mbox.troja.mff.cuni.cz; 4 Danubia NanoTech, s.r.o., Ilkovicova 3, 841 04 Bratislava, Slovakia; info@danubiananotech.com

**Keywords:** nanodiamonds, graphene oxide, *Escherichia coli*, antibacterial activity, inhibition

## Abstract

Nanodiamonds (NDs) and graphene oxide (GO) are modern carbon-based nanomaterials with promising features for the inhibition of microorganism growth ability. Here we compare the effects of nanodiamond and graphene oxide in both annealed (oxidized) and reduced (hydrogenated) forms in two types of cultivation media—Luria-Bertani (LB) and Mueller-Hinton (MH) broths. The comparison shows that the number of colony forming unit (CFU) of *Escherichia coli* is significantly lowered (45%) by all the nanomaterials in LB medium for at least 24 h against control. On the contrary, a significant long-term inhibition of *E. coli* growth (by 45%) in the MH medium is provided only by hydrogenated NDs terminated with C-H*_X_* groups. The use of salty agars did not enhance the inhibition effects of nanomaterials used, i.e. disruption of bacterial membrane or differences in ionic concentrations do not play any role in bactericidal effects of nanomaterials used. The specific role of the ND and GO on the enhancement of the oxidative stress of bacteria or possible wrapping bacteria by GO nanosheets, therefore isolating them from both the environment and nutrition was suggested. Analyses by infrared spectroscopy, photoelectron spectroscopy, scanning electron microscopy and dynamic light scattering corroborate these conclusions.

## 1. Introduction

Carbon nanomaterials such as nanodiamond (ND) and graphene oxide (GO) are considered highly promising for diverse biomedical applications such as long-lasting medical implants, bone tissue engineering, biosensors or drug delivery [1,2,3,4,5,6]. This is especially due to their beneficial chemical and physical properties such as general biocompatibility and non-toxicity, stable yet widely adjustable surface chemistry as well as suitable optical and electronic properties.

Even though these materials exhibit good biocompatibility, recent studies pointed out bactericidal properties of nanodiamonds and graphene oxide. It was shown that medical steel coated with a ND layer significantly suppresses the growth of the *E. coli* cells compared to pure medical steel or titanium surface without coating [7]. Another work stated that the bactericidal effect of NDs is most likely due to their partially oxidized surfaces where reactive oxygen-containing surface groups foster interactions of NDs with cellular components while the anisotropic distribution of charges on the ND surface facilitates alterations in bacterial surfaces [8]. A similar mechanism of antibacterial interaction between NDs and bacteria was also deduced in [9]. Our prior studies showed that NDs can modify different bacteria in a different manner. While *Escherichia coli* indicated a drop in the number of colonies compared to the reference due to the presence of NDs, *Bacillus subtilis* indicated a similar number of colonies but smaller colony sizes [10].

GO is also extensively proposed as an effective antibacterial agent in commercial product packaging and for various biomedical applications. Various works confirmed bactericidal properties of graphene-based materials, namely pristine graphene (G), graphene oxide (GO) and reduced graphene oxide (rGO) [11,12]. The suggested mechanism was different for various types of graphene-based materials. It was argued that superoxide anion generated by GO can disrupt the membrane of bacteria and that disruption of the cell membrane can also appear when it comes in direct contact with the sharp edges of GO nanowalls. Graphene nanosheets were also found able to be able to extract large amounts of phospholipids from the cell membranes due to strong interactions between graphene and lipid molecules [13].

On the other hand, our recent study of interaction of various NDs, GO or rGO with *E. coli* in Mueller Hinton (MH) broth and on MH agars showed that after 24 h the nanomaterials had no statistically significant antibacterial effect except for hydrogenated NDs that decreased the number of colony forming unit (CFU) by 50% [14]. Thus, there are pronounced differences in the reported degree of antibacterial effects as well as the proposed mechanisms. This is not surprising as various inorganic nanoparticles have been found to exhibit bactericidal properties and cause growth inhibition but the mechanisms of toxicity are generally not yet fully understood [15]. Most likely, it is due to different conditions in so far reported studies, such as specifically employed nanomaterial, a method of application (in the volume, on the surface) and also type of examined microorganism and culture medium.

In order to unambiguously elucidate the mechanism(s) of the antibacterial properties of ND or GO, one possible approach is to perform a comparison of the materials (with well-characterized properties) under the otherwise same conditions, such as in the case of cell interaction with graphene and nanocrystalline diamond thin films [3]. Therefore, in the present study, we compare bactericidal properties of well-characterized NDs, GO and rGO under the same conditions including nanomaterial and bacterial concentrations. We perform the comprehensive study in two different culture media, Luria-Bertani (LB) and Mueller-Hinton (MH) broths. Thereby we disclose the specific effect of cultivation media as well as we elaborate on the origin of antibacterial properties of the aforementioned nanomaterials. The results may be useful for prosthetics and implants as well as for water purification and preservation of its quality.

## 2. Materials and Methods

### 2.1. Materials

Four types of nanomaterials were employed in our study and mentioned in the further text. As-received NDs produced by detonation process are labelled as HND due to their numerous C-H bonds and positive zeta potential [16]. NDs that were annealed in air at 450 °C for 30 min to oxidize their structure [17], are labelled as OND. The nominal diameter of both types of NDs is 5 nm.

The third nanomaterial was graphene oxide (GO), which was produced by oxidation from graphite powder according to Bangal method [18], filtered through a nylon membrane and finally sonicated in an ultrasonic bath for 3 h. Estimated sizes of GO flakes is 0.5 to 2 μm. The fourth material, reduced graphene oxide (rGO) was produced by oxidation from graphite powder according to Brodie method [19], filtered through a nylon membrane, dried and thermally reduced in an argon atmosphere at 750 °C to obtain well-exfoliated rGO flakes. Estimated size of rGO flakes is 0.1 to 1 μm. More details about the employed GO and rGO materials can be found in [14].

**Figure 1:**
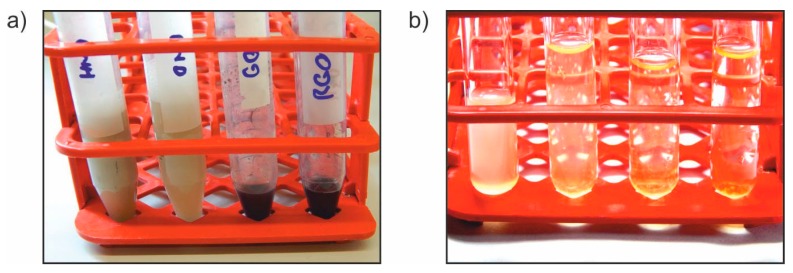
Photographs of (**a**) vials with HND, OND, GO and rGO (from the left to the right) stock dispersions (2 mg/mL) in distilled water and (**b**) vials with different concentrations of *E. coli* in the MH broth (from the left to the right decreasing relative concentrations related to the original one marked as 10^0^, 10^−1^, 10^−2^ and 10^−3^).

All nanomaterials used in this work were dispersed in distilled water to achieve a concentration of 2 mg/mL. Figure 1a shows a photograph illustrating appearance of such dispersed suspensions. The suspensions were homogenized in an ultrasonic bath for 30 min and sterilized by autoclave at 120 °C. We have confirmed that the sterilization in autoclave did not affect surface chemistries of NDs and GO/rGO sheets [14]. Figure 1b illustrates decreasing turbidity of suspension with *E. coli* due to increasing dilution in the MH broth (samples in LB broth look similarly).

### 2.2. Microbiological Studies

The microbiological study was performed in two different media—Mueller-Hinton (ready made powder purchased from the Oxoid Ltd., Basingstoke, UK) and Luria-Bertani (powder components purchased from the Oxoid Ltd., Basingstoke, UK) in both liquid and solid phase: Mueller-Hinton broth (MHB) and agar (MHA), Luria-Bertani broth (LBB) and agar (LBA). The MH broth was prepared according to the instructions of the supplier by mixing of 21 g of ready-made MH powder with 1 L of distilled water. MH broth prepared by this way contains beef infusion, casein hydrolysate and starch [20]. The LB broth was prepared according to the recipe [21] by mixing of distilled water (1 L) with tryptone (10 g), yeast extract (5 g) and sodium chloride (10 g). The pH was balanced to 7.2 ± 0.2 in both cases. The agars were prepared from the broth liquids by the addition of 20 g of agar powder per 1 L of the broth.

The sample of bacterial suspension was prepared as follows: We spread 1 mL of concentrated *E. coli* suspension (taken from the freezer and melted for 15 min) on 9 cm Petri dishes with the agar (either MHA or LBA) and let it grow overnight in the thermostat at the temperature of 37 °C. Then all the bacteria from the Petri dish were wiped off and put into 5 mL of a broth (either MHB or LBB). This concentration of the bacteria was unity and marked as 10^0^.

The initial bacterial suspension was diluted by the broth in several steps with dilution ratio 1:10 in each step. Figure 1b shows a photograph illustrating the appearance of the suspensions with different bacterial concentrations. We stopped diluting of the suspension when its turbidity, around 0.5 MFU (MacFarland Units), was achieved. The closest value to this turbidity had a sample with relative concentration of 10^−3^ (turbidity of 0.4 MFU in case of both MHB and LBB). This bacterial concentration was used in all the performed experiments.

Then we prepared five test tubes for each broth type. Four of them were filled with 3 mL of bacterial suspension in appropriate broth and 3 mL of the suspension with the examined material. Thereby the concentration of examined nanomaterials was 1 mg/mL. The fifth test tube was a reference one, in which 3 mL of bacterial suspension were diluted by 3 mL of distilled water.

All test tubes were put into the shaker inside of the thermostat set at 37 °C. The first set of samples was taken from each tube after 5 h to examine the exponential growth phase of bacteria. Each sample was then gradually diluted to the relative concentration 10^−10^ while the original concentration taken from each test tube was 10^−3^. We used concentrations 10^−9^ and 10^−10^ for the further cultivation on Petri dishes.

These concentrations of each material were spread on three 9 cm Petri dishes in a triplet with the agar which represented a set of 15 Petri dishes for each type of media. The amount of the spread suspension was always 1 mL. Then we put all Petri dishes into the thermostat set at 37 °C for 24 h.

Another set of samples was taken from test tubes with broth after 24-h of incubation to examine the stationary phase performing the same dilution, spreading and cultivation procedure like we did with the 5 h set. The original concentration taken from each tube was still considered 10^−3^. The bacterial cultivation on agar plates (MHA or LBA), either original or enhanced by the addition of 40 g/L NaCl, was also performed for 24 h at 37 °C.

The bacterial colonies on Petri dishes were then counted and the average number of colonies was compared with the negative control sample (100% = the average for the control sample). The two-sample *t*-test for unequal variance with six participants in each group was used for the evaluation and comparison of colony unit counts against reference. There were two various bacteria concentrations in triplicates for each sample, which were recalculated to unity concentration.

We also performed an experiment with salty agars. To obtain salty agars we prepared MH and LB agars in the usual way and finally, we added sodium chloride into the agar liquid in a concentration of 40 g/L. Then we compared the growth of bacteria on normal and salty agars to find whether the combination of salt and nanomaterials would result in a synergic stress effect on the bacteria.

### 2.3. Material Analytical Techniques

Surface chemistry of the LB and MH broths was characterized by attenuated total reflectance Fourier transform infrared (ATR-FTIR) spectroscopy. Details about the spectrometer, its accessories and evaluation method can be found in [14]. In all cases, the spectra represent an average of 128 scans recorded with a resuspension of 4 cm^−1^. Spectra were normalized at 1632 cm^−1^ (1590 cm^−1^ resp., AMID I band). Advanced ATR correction was applied on all measured spectra.

X-ray photo electron spectroscopy (XPS) analysis of the broth samples of MH and LB was performed on AXIS Supra (Kratos Analytical Ltd., Manchester, UK) using monochromated Al Kα X-ray source (1486.6 eV) and a hemispherical energy analyser (analysed area—0.7 × 0.3 mm^2^). XPS photoelectron survey spectra were acquired at a constant take-off angle of 90° using 80 eV pass energy. Samples were prepared by deposition of 100 µL broth suspension on an Au/Si substrate. After drying in a stream of nitrogen, the samples were further dried in a vacuum of 10^−5^ mbar for 2 days.

The size distribution and ζ-potential of the NDs, GO, rGO and *E. coli* colloidal suspensions were measured by dynamic light scattering (DLS) at 25 °C using a Nano-ZS (Malvern Instruments Ltd., Malvern, UK) equipped with HeNe laser. The disposable folded capillary cell was used to eliminate sample cross-contamination. The samples were not filtered nor centrifuged prior to the DLS measurements.

Scanning electron microscopy of the bacteria was performed by FE-SEM Mira 3 microscope (Tescan Brno s.r.o., Brno, Czech Republic) at an electron beam energy of 30 keV. The top-view micrographs were acquired using an in-beam detector in the secondary electrons mode at a working distance of about 5 mm. The bacteria were sampled directly from LB suspension with nanomaterials, diluted 100× in water and drop casted on a rough side of Si wafer substrate. The samples were consequently dried in air for 5 min.

## 3. Results

### 3.1. Microbiological Studies

We investigated biological effects of nanodiamond and graphene particles on the *E. coli* strain CCM 3954 (Czech Collection of Microorganisms). The bacteria were tested in two phases of the growth curve; the exponential phase (5 h exposition) and stationary phase (24 h exposition). The doubling time is constant during the exponential phase while the number of bacteria remains constant during the stationary phase. We cultivated a mixture of diluted *E. coli* suspension in the MHB or LBB with the suspension of examined materials (HND, OND, GO and rGO).

**Figure 2:**
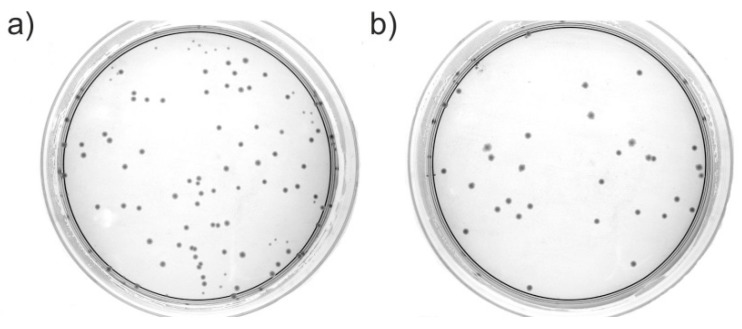
Comparison of Petri dishes cultured using (**a**) the *E. coli* reference sample and (**b**) the *E. coli* sample exposed to HND in the LB medium for 5 h.

Figure 2 shows a comparison between the *E. coli* reference sample and the sample where *E. coli* was exposed to HND in the LB medium for 5 h. More examples of Petri dishes for LB an MH media after 5 or 24 h are shown in the Supplementary information (Figure S1). We calculated the surviving ratio by counting the bacterial colonies (CFU) on the Petri dishes against the reference.

**Figure 3:**
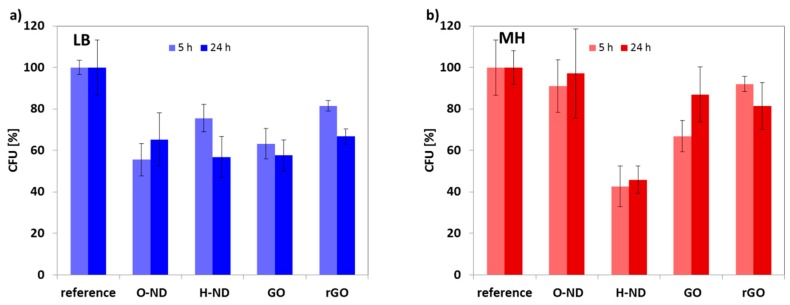
Effect of as received nanodiamond (HND), air annealed nanodiamond (OND), graphene oxide sheets (GO) and reduced graphene oxide sheets (rGO) on the number of colony forming unit (CFU) of *E. coli* after 5 and 24 h of incubation in two different media—(**a**) Luria Bertani (LB) and (**b**) Mueller Hinton (MH). Error bars represent the standard deviation of the mean values obtained from multiple experiments.

Figure 3 shows the bar graphs summarizing the bacteria surviving ratio after 5-h and 24-h exposure to ND, GO and rGO nanomaterials in both types of media. Error bars represent the standard deviation.

In the MH medium, the most evident microbiological response in the exponential phase was for HND reducing the number of CFU by 60% and for GO reducing the number of CFU by 35% after 5 h. The differences between the reference and HNDs or GO were evaluated at the significance level α = 0.005. The microbiological response to the OND and rGO was not statistically significant for the MH media. The only significant biological response after 24 h (stationary phase) for the MH was observed in case of HND reducing the number of CFU by 55%. The difference between the reference and HNDs was evaluated at the significance level α = 0.01 in this case.

In the LB medium, the most evident microbiological response in the exponential phase was for OND, which reduced the number of CFU by 45% after 5 h. In the case of this cultivation media, all other tested materials (HND, GO and rGO) show also a statistically significant response, where the reduction ration varies between 20% and 35%. The response in the stationary phase is very similar for all tested materials reducing the CFUs by 35% to 40%. The difference between the reference and each of materials was evaluated at the significance level α = 0.02 for 5 h measurement and at the significance level α = 0.01 for 24 h measurement.

Note that all these significance levels were below typically employed threshold α = 0.05. When α was obtained above this threshold, the differences were taken as not statistically significant.

**Figure 4:**
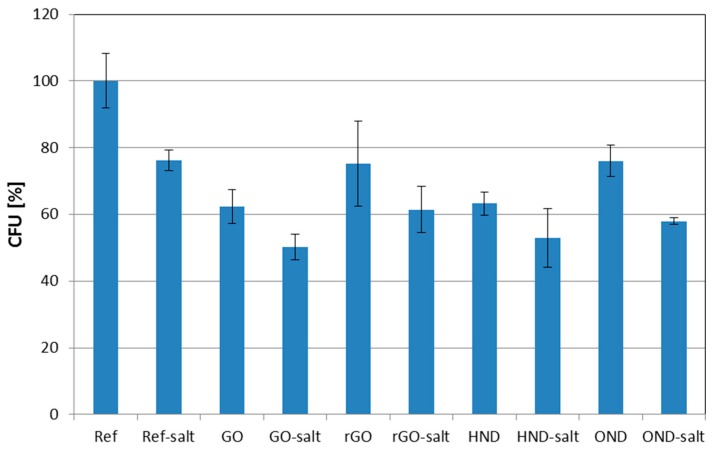
Effect of cultivation on salty agars on the number of CFU after 24 h of exposure to nanomaterials in the LB medium.

Experiments, where salty agars were used for *E. coli* cultivation after growing in the broth with nanomaterial exposure, did not prove any significant enhancement of the antibacterial effects of nanodiamonds and GO/rGO. Figure 4 summarizes the resulting inhibition of *E. coli* growth (CFU reduction) for all the materials after 24 h exposure in the LB broth.

Figure 4 shows the graph summarizing the effect of chosen nanomaterial on the bacterial growth enhanced by the use of salty agar. No evidence of antibacterial effect potentiation in medium enriched by Na^+^ and Cl^−^ ions has been recorded.

**Figure 5:**
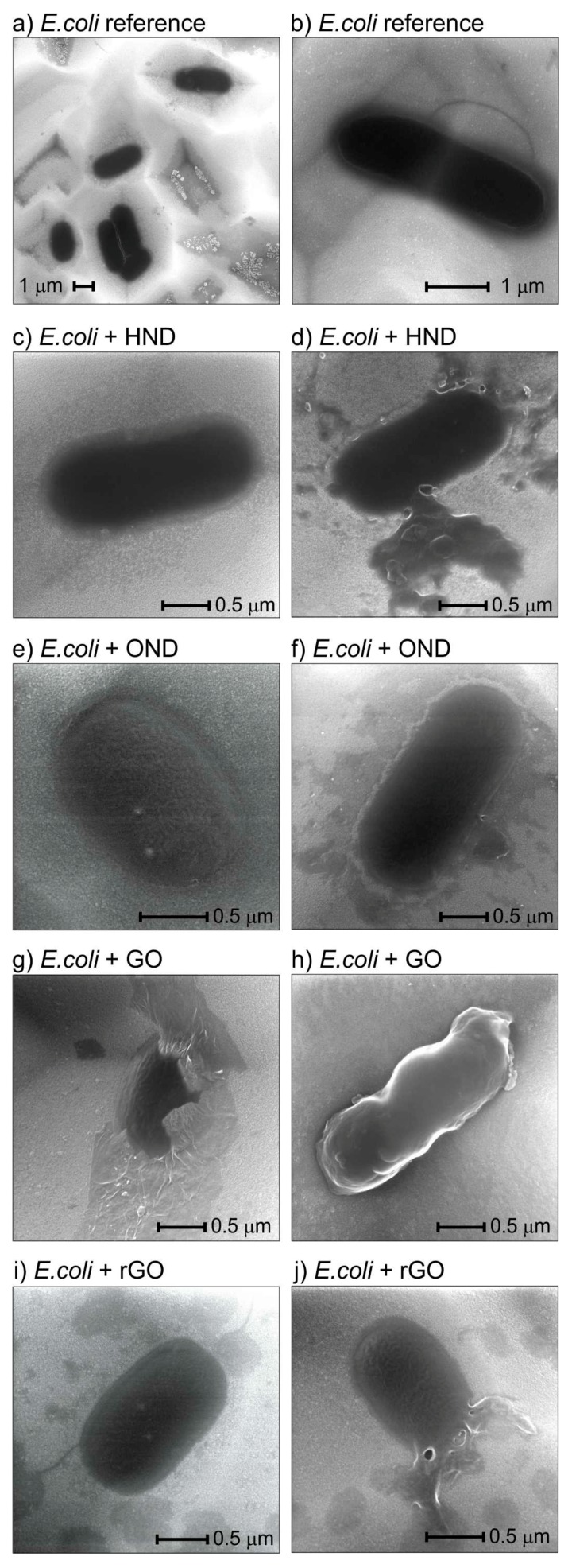
Typical SEM morphologies of *E. coli* sampled on silicon substrates from the reference suspension (**a**,**b**) and from suspensions where *E. coli* was exposed to HND (**c**,**d**), OND (**e**,**f**), GO (**g**,**h**), rGO (**i**,**j**). Set of two images for each material illustrates various morphologies of the samples.

Figure 5 shows SEM analysis of bacteria morphologies after interaction with nanomaterials in the LB broth. Overview SEM image on the sample with *E. coli* from the reference suspension in Figure 5a shows characteristic features found also on other samples: (i) bright assemblies of dots that correspond to salt crystals from the dried broth; (ii) grey regions around those dots or present also alone that correspond to other carbon remnants of the broth; (iii) dark round bodies of bacteria. For detailed morphology analysis, we focused on regions where broth remnants do not obscure images of bacteria. Figure 5b reveals a detail view of *E. coli* taken from the reference sample in which the rounded morphology is a characteristic feature of healthy *E. coli*. A protein fibril extending from the bacterium is also noticeable. In the morphology of *E. coli* after its incubation in the LB broth with HND there is noticeable a pronounced flat rim (Figure 5c) as well as rugged edges and leak of cytoplasm (Figure 5d). Upon magnification, one can resolve a powder decorating the bacteria edges—those are most likely the nanodiamond aggregates. Similar features are observed also on *E. coli* from the LB broth with OND (Figure 5e,f). For GO we observed most of the bacteria covered by GO sheets (Figure 5g) or some rugged bacteria (Figure 5h). Only thin flakes of rGO were observed around the bacteria (Figure 5i). Leaked cytoplasm was observed on rGO samples as well (Figure 5j) but not on GO samples.

One should critically note that the observed nanomaterial coverage on or around bacteria could be also at least partially due to mere adsorption during sample drop casting as all residual materials from the bacterial medium could not be removed with certainty. Also, note that the leaks must have inherently occurred on the substrate and not in suspension; otherwise they would not be visible. Thus, they most likely occurred due to membrane stress during deposition of bacteria on the substrate. The leaks might be due to the weakening of bacterial membrane by some of the nanomaterials (they were not observed for reference and GO). However, even if it is the case it was not significantly strong to enhance CFA reduction on salty agars.

Another observed feature is a wrinkled surface of bacteria exposed to OND, GO or rGO. However, such wrinkled membrane was observed in several instances also on the reference sample. Thus, it cannot be unambiguously interpreted as specific to OND, GO or rGO. It seems to be related to the bacteria drying process under vacuum in SEM. We did not notice disruptions of the bacterial membrane such as cutting, unlike in some prior reports [11,12]. This corroborates the results of experiments on salty agars.

Noticeable is also different size and shape of bacteria on each sample. We have analysed the bacterial dimensions in detail. Table 1 summarizes average length and width of *E. coli* as measured from sets of SEM images obtained on all samples. The average ratio of length to width (*L*/*W*) is also provided. The statistically significant difference (*p* value < 0.05) of the size ratio to other samples is indicated by sample numbers. Bacteria size and shape do not differ significantly between the reference and HND samples. For OND, GO and rGO samples the bacteria grow significantly shorter though. This indicates the different antibacterial mechanism of HND compared to the other nanomaterials. It also corroborates the result that HND effect is more or less independent of LB or MH broth while the effect of other nanomaterials is significant only in the LB broth.

### 3.2. Media Characterization

The FTIR spectroscopy did not reveal specific differences between the two employed culture media. Both spectra revealed just general spectroscopic features of peptide/protein chains [22]. The only significant difference can be seen in the shape of the spectral band at 1700−1500 cm^−1^. This band consists of several overlapping bands. Its different shape may reflect various secondary structures of peptides/proteins in the examined media. Comparison of the ATR FTIR spectra of MH and LB broths is shown and described in more detail in the Supplementary information (Figure S2).

The XPS analysis was also performed to evaluate any significant differences in compositions or possible elemental contaminations (such as bactericidal silver or copper) of LB and MH broths. Contamination of the media could weaken the bacteria so that they would react more sensitively to additional stress introduced by nanomaterials. However, results summarized in Table 2 show very similar atomic compositions for both media and no contaminating elements. Expected presence of Ca^2+^ and Mg^2+^ ions usually found at 347.2 eV (Ca 2p) or above 1303 eV (Mg metal 1303 eV, Mg native oxide 1304.5 eV, MgCO_3_ 1305 eV) [23] was not detected in the wide range survey scans. The content of these ions is presumably under the detection limit of our XPS setup.

DLS results revealed pronounced changes in the distribution of colloidal particle size. In the case of nanodiamond can observe the formation of approximately ten times larger “particles” (actually nanomaterial aggregates) in broths (LB and MH) compared to water. In the case of GO, there was a significant shift to larger particles caused by broths as for nanodiamonds but there was also the noticeable formation of ten times smaller particles compared to GO dispersion in water. This is in accordance with prior observations in the literature [10,24]. All DLS graphs are summarized in the Supplementary information (Figure S3). We also measured ζ-potentials of the materials in water and bacterial culture media. The results are summarized in Table 3. Each value is an average of 9 measurements rounded to the integral number. All values have a statistical error of about ±2 mV.

In case of the GO the absolute value of ζ-potential slightly decreases in LB and MH media, however, the values remain in the same stability range. On the other hand, the dilution in broth causes a significant drop in the magnitude of ζ-potential in nanodiamond solutions, which shifts the colloid to the unstable range, especially in case of the HND. A similar shift to the unstable range was observed in case of *E. coli* dilution in broth compared to dilution in water. Quite surprising was the change in polarity of the HND ζ-potential in broths compared to water. This may indicate the formation of pronounced protein corona around the particles. Although other materials already have negative ζ-potential, a decrease to less negative values in cell culture media is noticeable in all cases. Difference between zeta potential of nanomaterials between LB and MH is most likely caused by adsorption of different molecules and ions due to different composition of the two media. While the LB medium contains tryptone, yeast extract and salt [21], the MH medium contains beef extract, casein hydrolysate and starch [20].

## 4. Discussion

Antibacterial effects of the employed carbon nanomaterials are significantly different in the specific media. While only HND exhibited significant reduction ratio of bacterial CFU in the MH medium, there was uniform reduction ratio in the LB medium across all the materials. The difference is probably caused by the presence of more stressors for the bacteria in case of the LB medium supported by the presence of nanomaterials and their antibacterial activity.

Since we tested a set of microorganisms, we had to keep in mind that negative influence on individual bacterium could be compensated by reaction of other bacteria. This surviving strategy is limited by stress factors. That is why the testing of biological properties of nanomaterials should include also analysis of stressors. In real conditions, we can expect the presence of several stressors (physical, chemical, nutritional).

There are two basic modes of action of nanomaterials affecting the *E. coli* growth: direct mechanical interaction and/or oxidative stress [25]. Accumulation of nanoparticles in close vicinity of bacteria can be caused by the electrostatic interaction of nanoparticles with (typically negatively) charged cellular surface. Inducted membrane stress can result in lethal changes of the cell structure [26]. Negatively charged domains of bacterial flagella proteins can attract positively charged nanoparticles [27]. Accumulated nanoparticles can form either thin layers around cells (in case of GO) or large aggregated particle clusters (rGO and NDs) [10,24]. Consequential intimate contact with nanoparticles can cause modification of cell vital structures by local chemical or electrostatic interaction [24]. Spectroscopic signatures obtained from biomolecules such as adenine and proteins from bacterial cultures with different concentrations of GO, were used to probe the antibacterial activity of GO at the molecular level. The observation of higher intensity Raman peaks from adenine and proteins in GO treated *E. coli* correlated with induced death. The antibacterial action of GO was thus related to disruption of the cell membrane by GO [13].

However, in our case, the values of ζ-potential of carbon nanomaterials and bacteria in cell culture media are all negative. Thus, direct electrostatic attraction of nanomaterials to bacteria can be excluded. In the LB, ζ-potential values of nanomaterials are quite comparable (considering also the statistical error), between −15 mV to −28 mV, not correlated with the bacterial growth inhibition trend. There are more pronounced ζ-potential differences of nanomaterials in MH, between −7 mV to −31 mV, yet again, there is no correlation with the inhibition trend. The antibacterial effect cannot be explained just in regard to the change of ζ-potential. The electrokinetic surface properties of nanomaterials or the electric interaction between the bacterial membrane and nanomaterial is therefore probably not the sole negative action of nanomaterial responsible for bacterial growth inhibition.

The nanomaterials may not attach and interact with the bacteria surface only electrostatically though. It can occur on the mechanical basis or by other chemical interactions. During the exposure to nanomaterials in the media, the bacterial culture is continuously agitated on a shaker plate. Thereby the mutual interaction is promoted, with or even without permanent nanomaterial attachment to the bacteria surface. To what degree are the bacteria coated or not by the nanomaterials is in our case still not clear though. Unfortunately, it was technically impossible to image unambiguously nanomaterial coverage on bacteria by optical or electron microscopy as we could not remove all residual materials in the culture medium.

Nevertheless, the results of *E. coli* cultivation on salty agars showed no further enhancement of antibacterial effect. Thus, disruption of bacterial membrane or difference in ionic concentration are not key factors behind the antibacterial effect and observed differences for specific nanomaterials and media.

Oxidative stress is an additional basic mechanism of nanomaterial toxicity. Superoxide anion (O^2−^) is produced as a by-product of oxygen metabolism and, if not regulated, causes many types of cell damage. The exposure to nanomaterial is connected with the generation of reactive oxygen species (ROS), which are thought to be responsible for the bactericidal effects of many inorganic nanoparticles (e.g., Ag, Cu, MgO, ZnO, CeO_2_, TiO_2_, Al_2_O_3_ demonstrated on *B. subtilis*, *E. coli*, *P. aeruginosa*, *E. faecalis* and *S. aureus*, to name just a few) [15]. However, the quantitative relationship between ROS activity and antibacterial activity have not been established so far. The factors for nanomaterial-induced oxidative stress in bacteria are many as it is a complex system in the oxidative metabolizing organism and therefore is influenced not only by the size of nanomaterial but also chemical composition of nanoparticle and its purity, surface charge, coatings and functionalization as well as band gap energy and illumination.

The role of hydroxyl radical abundance was excluded in pure rGO suspensions [28] and superoxide anion abundance [24] was excluded in GOs suspensions. In vitro glutathione (GSH) oxidation induced by the presence of graphite, graphite oxide, GO and rGO in suspensions showed that the GSH can be used as a perspective marker of general oxidative stress independent on particular reactive oxygen species detection [24].

Previous study of the antibacterial activity of GO, rGO and other graphite materials towards *E. coli* under similar concentrations and incubation conditions showed that GO dispersion exhibits the highest antibacterial activity, followed by rGO [24]. No reactive oxygen species production was detected though. However, it was argued that GO and rGO materials can oxidize glutathione, which serves as redox state mediator in bacteria.

NDs were reported to increase superoxide dismutase (SOD) activity and at the same time decreased the activity of glutathione reductase (GR) and glutathione peroxidase (GPx) within erythrocytes [29]. NDs did not significantly affect either the total antioxidative state (TAS) nor the thiobarbituric acid reactive substances (TBARS) in blood plasma.

Thus, oxidative stress seems to be the dominating factor behind the antibacterial effect of nanodiamonds (where it may be further enhanced by pronounced internalization) and graphene oxide. On the other hand, there is still remaining question is: Why does the LB broth enhance the effect of GO and rGO? The principal difference between the MH and LB cultivation media is in the concentration of the Mg^2+^ and Ca^2+^ ions. The concentration of these ions is several times higher in case of the MH media compared to the LB ones [30]. These ions are needed to bridge the highly negatively charged lipopolysaccharide molecules forming the outer membrane of the *E. coli*. The absence of essential cations necessary for enzymatic biochemical reactions is an effective stressor for augmentation of nanomaterial effect on the microorganism growth. In addition, deficiency of Mg^2+^ and Ca^2+^ ions has been correlated with increased oxidative stress signalled by increased superoxide anions in blood plasma [31]. Another limiting factor for the LB media in comparison with MH media is consumption of utilizable nutrients. Unlike MH media where starch is a key component, LB media provides only a scant amount of carbohydrates. The dominant LB media nutrients source is tryptone and yeast extract peptides. However, utilization of these peptides is limited by a molecule size of approximately 650 daltons (peptides must be transferred into cells through porins). Consequently, the metabolism of cells is limited by consumption of easily utilizable amino acids—*E. coli* growth behaviour goes through diauxie-like behaviour—and then available but hardly utilizable amino acids are used. This fact changes both growth rate and vulnerability of cells [21].

A recent study suggested that the antibacterial activity of nanodiamond is linked to the presence of partially oxidized and negatively charged surfaces, specifically those containing acid anhydride groups [8]. Furthermore, proteins were found to reduce the bactericidal properties of nanodiamonds by their covering with such surface groups. Our data do not confirm these assertions. Hydrogenated nanodiamonds acted here as the universal material, the most pronounced antibacterial agent in both LB and MH media, in spite of obvious encapsulation by proteins and a related in its ζ-potential to negative. Air annealed (oxidized) nanodiamonds with similar negative ζ-potential had only limited effect, even less than GO or rGO in the MH broth.

## 5. Conclusions

We investigated the changes of bacteria growth in suspension caused by the presence of nanodiamonds and graphene oxide in two types of media—MH and LB. Our findings showed that the effect of nanomaterial presence is more pronounced and uniform in the LB media, where all nanomaterials had a similar reduction ratio of the CFU number. The effect was long-term and persists for at least 24 h. On the contrary, the antibacterial effect of nanomaterials in the MH media was significant only in the case of the HND. The antibacterial effect of the employed carbon nanomaterials was enhanced in the LB medium and it was suggested that the absence of Ca^2+^ and Mg^2+^ ions comparing to MH medium might be of the main importance besides the difference in the amount of carbohydrates. The mechanism was not related to the disruption of bacterial membrane or differences in Na^+^ and Cl^−^ ionic concentrations as the inhibition effects were not synergistically enhanced on salty agars. Only additive or insignificant effect was observed when the bacteria were cultured on salty agars. The mechanism can thus be attributed to oxidative stress induced by the presence of nanodiamonds or graphene oxide in the broths. The enhanced sensitivity of *E. coli* to GO and rGO in LB medium is most likely related to deficiency of Mg^2+^ and Ca^2+^ ions. But the oxidative stress will not be the sole toxic mode of action of nanodiamond as HNDs (unlike ONDs) seemed to have similar antibacterial properties. These findings open prospects for bactericidal treatments of liquids mainly concerning their Mg^2+^ and Ca^2+^ ion concentration by these carbon nanomaterials. Possible future applications of carbon nanomaterials are long-lasting medical implants, bone tissue engineering, biosensors or drug delivery [5,6].

## Figures and Tables

**Table 1:** Average length and width of *E. coli* as measured from sets of SEM images. The average ratio of length to width is also provided. The statistically significant difference (α < 0.05) of the size ratio to other samples is indicated by sample numbers. Sdev denotes the standard deviation of the average values.

No#	Bacteria Sample	Length (nm)	Sdev-L (nm)	Width (nm)	Sdev-W (nm)	Ratio *L*/*W*	Sdev	*Significant Difference*
1	*E. coli* ref.	2757	723	1017	92	2.74	0.78	*3,4,5*
2	*E. coli* + HND	2511	632	1050	101	2.42	0.70	*3,4*
3	*E. coli* + OND	1685	278	1002	97	1.68	0.24	*1,2*
4	*E. coli + GO*	1730	443	976	173	1.79	0.46	*1,2*
5	*E. coli* + rGO	1587	447	864	102	1.87	0.64	*1*

**Table 2:** Elemental compositions of the bacterial culture media.

Medium	O, at.%	C, at.%	N, at.%	Na, at.%	Cl, at.%	S, at.%
MH broth	21	65	11	1	1	1
LB broth	23	63	11	1	1	1

**Table 3:** Measured ζ-potential of the materials in water and bacterial culture media. All values have a statistical error of ±2 mV.

	Zeta Potential (mV)				
Material	OND	HND	GO	rGO	*E. coli*
H_2_O	−38	+39	−37	−37	−26
LB	−15	−18	−28	−22	−9
MH	−10	−7	−31	N/A	−8

## Supplementary Materials

The following are available online at http://www.mdpi.com/2079-4991/8/3/140/s1.
