# Supplementary material for: Inhibition of E. coli Growth by Nanodiamond and Graphene Oxide Enhanced by Luria-Bertani Medium
Source: Nanomaterials (Basel). 2018 Mar 1;8(3):140. doi: 10.3390/nano8030140 (PMC5869631; doi:10.3390/nano8030140)
Supplement: Supplementary file 1 [file nanomaterials-08-00140-s001.docx]

Supporting Online Material for

**Inhibition of E. coli growth by nanodiamond and graphene oxide**

**enhanced by Luria-Bertani medium**

Jaroslav Jira^1,2^ *, Bohuslav Rezek^2^, Vitezslav Kriha^2^, Anna Artemenko^1^, Iva Matolínová^3^, Viera Skakalova^4^, Pavla Stenclova^1^ and Alexander Kromka^1^

^1^ Institute of Physics, Academy of Sciences of the Czech Republic, Cukrovarnická 10, 162 00 Prague 6, Czech Republic; kromka@fzu.cz

^2^ Faculty of Electrical Engineering, Czech Technical University, Technická 2, 166 27 Prague 6, Czech Republic; rezekboh@fel.cvut.cz

^3^ Faculty of Mathematics and Physics, Charles University, V Holešovičkách 2, 18100 Prague 8, Czech Republic;

imatol@mbox.troja.mff.cuni.cz

^4^ Danubia NanoTech, s.r.o., Ilkovicova 3, 841 04 Bratislava, Slovak Republic

info@danubiananotech.com

* Correspondence: jira@fel.cvut.cz , Tel: +420-603-893118

Contents

[1 Supplementary 1: Examples of Petri dishes with grown colonies 2](#_Toc490144279)

[2 Supplementary 2: FTIR analysis - comparison of the ATR FTIR spectra of MH and LB broths 4](#_Toc490144280)

[3 Supplementary 3: DLS analysis 5](#_Toc490144281)

# **Supplementary 1: Examples of Petri dishes** with grown colonies


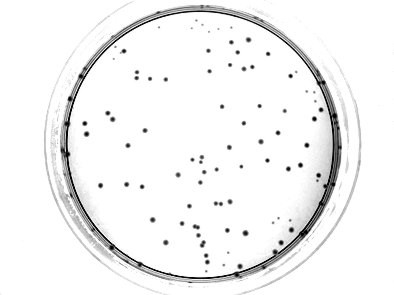

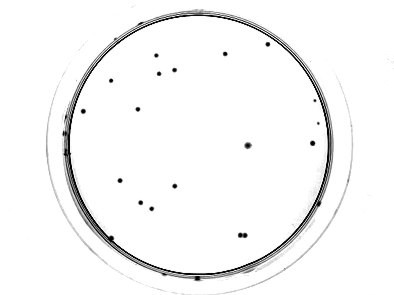


MH 5 hours ref-HND


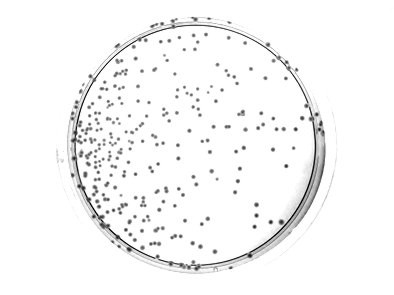

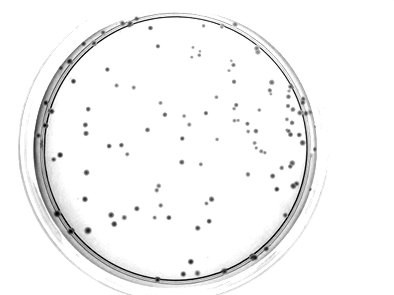


MH 24 hours ref-HND


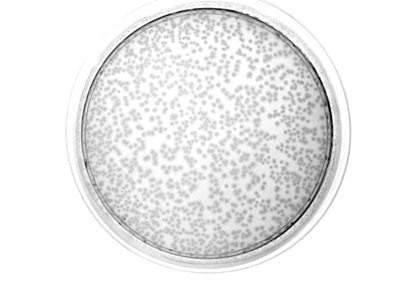

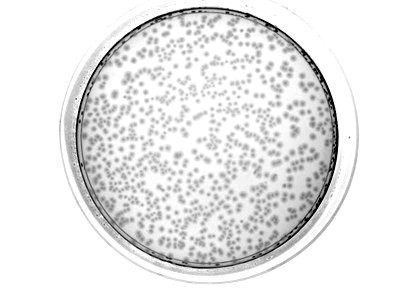


LB 5 hours ref-OND


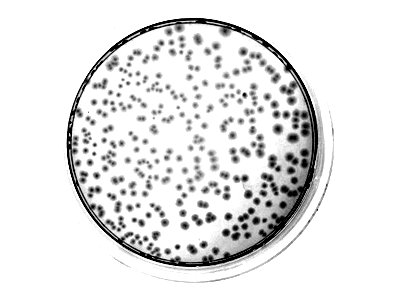

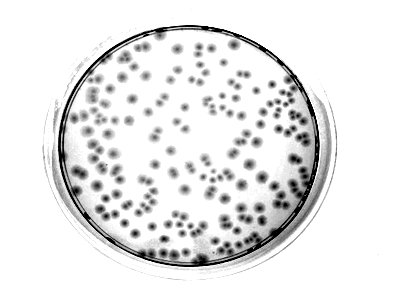


LB 24 hours ref-HND


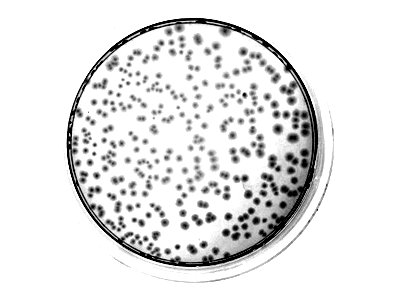

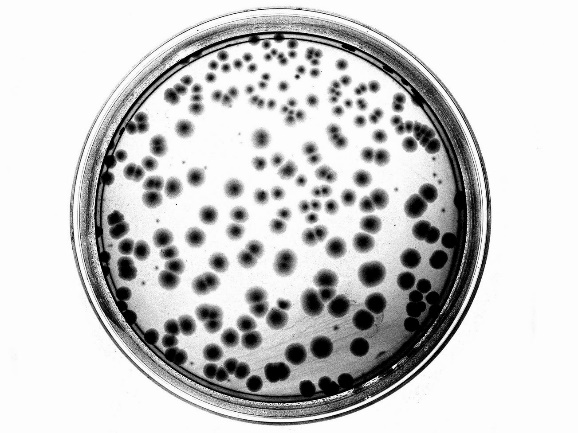


LB 24 hours ref-GO


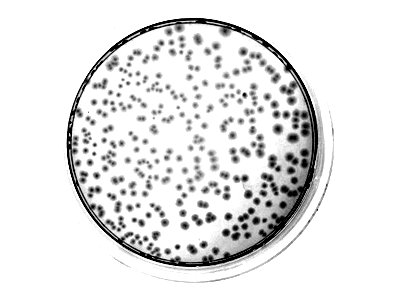

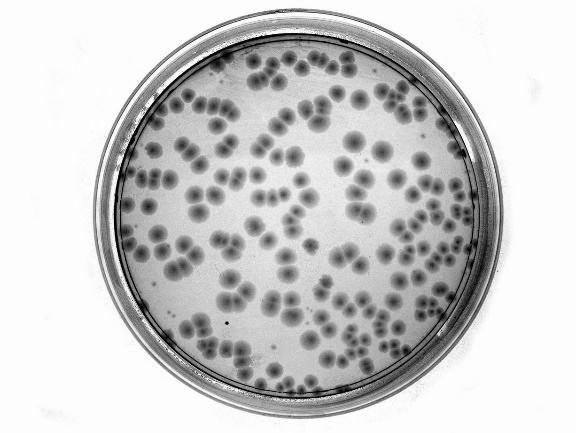


LB 24 hours ref-rGO

**Figure S1.** Examples of Petri dishes with grown colonies. Comparison between the reference sample and samples with nanodiamonds and graphene oxide sheets are shown. Images were converted to grayscale and contrast adjusted for better visibility.

| **Supplementary 2: FTIR analysis** - comparison of the ATR FTIR spectra of MH and LB broths 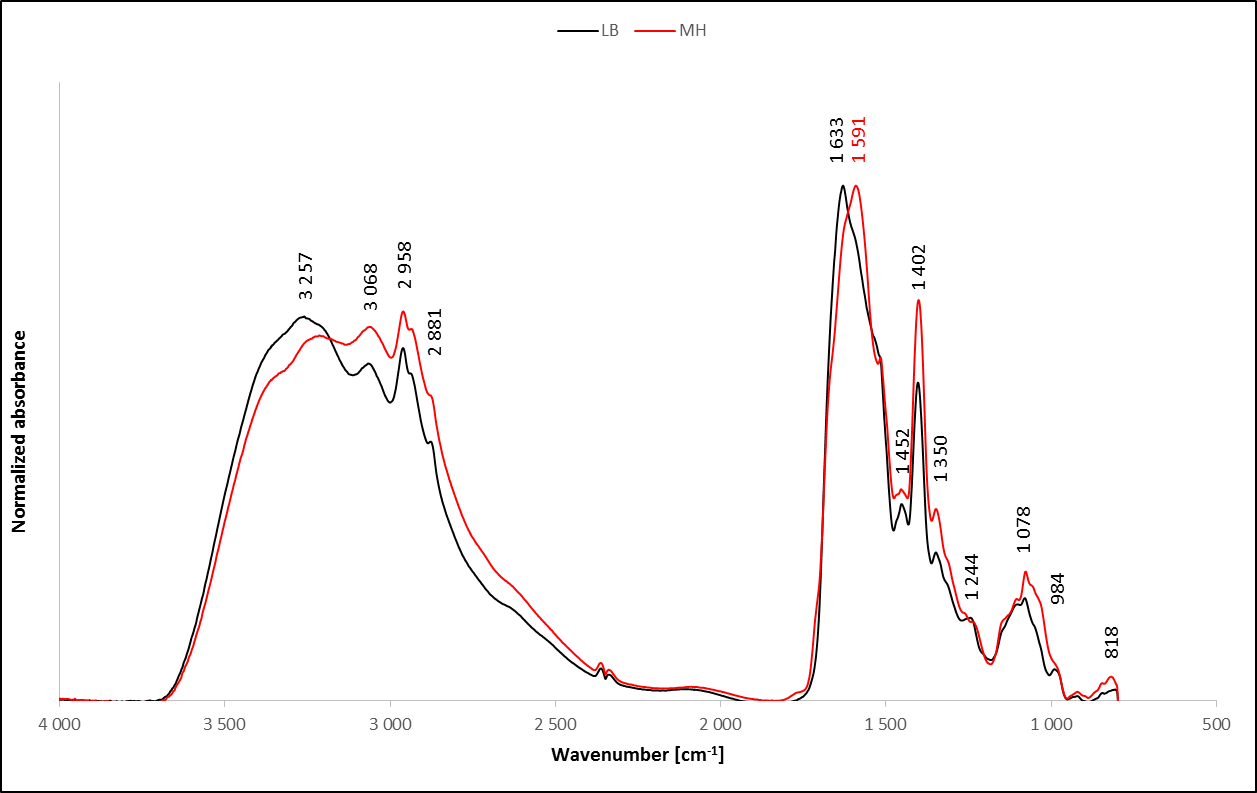 |  |
| --- | --- |
| **Figure S2.** ATR FTIR spectra of the LB and MH broths. | |

FTIR spectra of both LB and MH are dominant by a broad absorption band at 3200 cm^−1^ related to the stretching of OH groups. Sharp bands at 2961, 2963 and 2874 cm^−1^ are related to stretching of aliphatic CH_x_ groups. Moreover, one can distinguish bands at 3254 cm^−1^ and 3063 cm^−1^ from AMID A and AMID B, respectively. This so-called Fermi doublet is typical for proteins. Additionally to the AMID B band, the band at 3063 cm^−1^ can be also contributed by stretching of aromatic CH in protein side chains.

The area below 1700 cm^−1^ is dominating by AMID I (1632 cm^−1^ C=O stretching) and AMID II (shoulder at 1523 cm^−1^, N–H stretch) bands confirming the presence of proteins. The strong sharp band at 1404 is attributed to vibrations within the COO^−^ groups. Bands of medium intensity at 1453, 1346 a 1242 cm^−1^ are related to various CH_x_ bending vibrations. Maxima at 1244 and 1078 cm^−1^ can be attributed to asymmetric and symmetric stretching of PO^2−^ groups from phosphates naturally found in yeast extract or nucleotides [1], [2]. Nevertheless, the shape of maximum at 1078 cm^−1^ can indicate overlap of additional bands of alcohols or various CH_x_ groups.

**References**

1. Teleman, A.; Richard, P.; Toivari, M.; Penttilä, M. Identification and Quantitation of Phosphorus Metabolites in Yeast Neutral PH Extracts by Nuclear Magnetic Resonance Spectroscopy. *Anal. Biochem.* **1999**, *272* (1), 71–79 , DOI: 10.1006/abio.1999.4165.

2. Barth, A. Infrared Spectroscopy of Proteins. *Biochim. Biophys. Acta BBA - Bioenerg.* **2007**, *1767* (9), 1073–1101 , DOI: 10.1016/j.bbabio.2007.06.004.

# Supplementary 3: DLS analysis


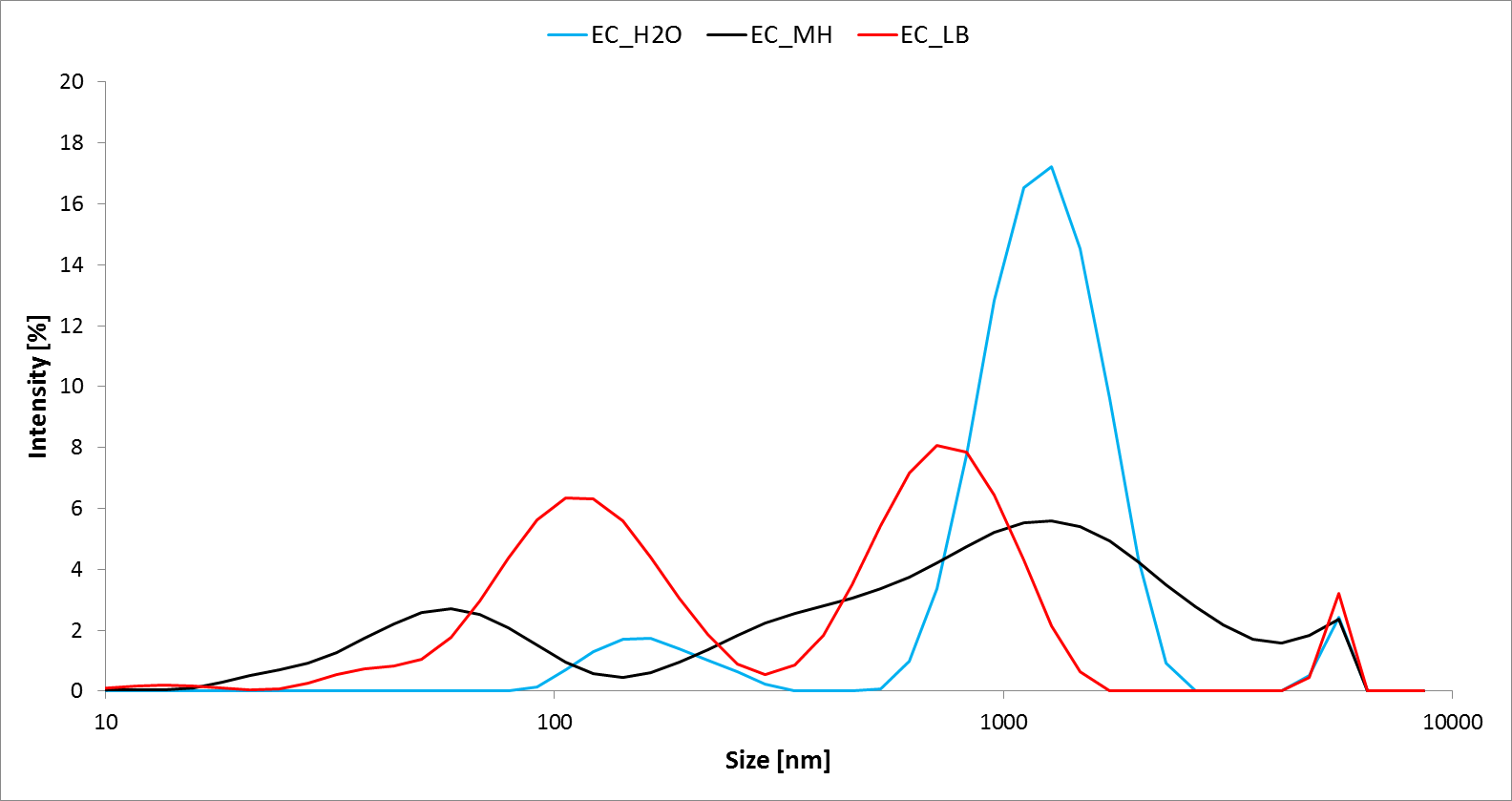


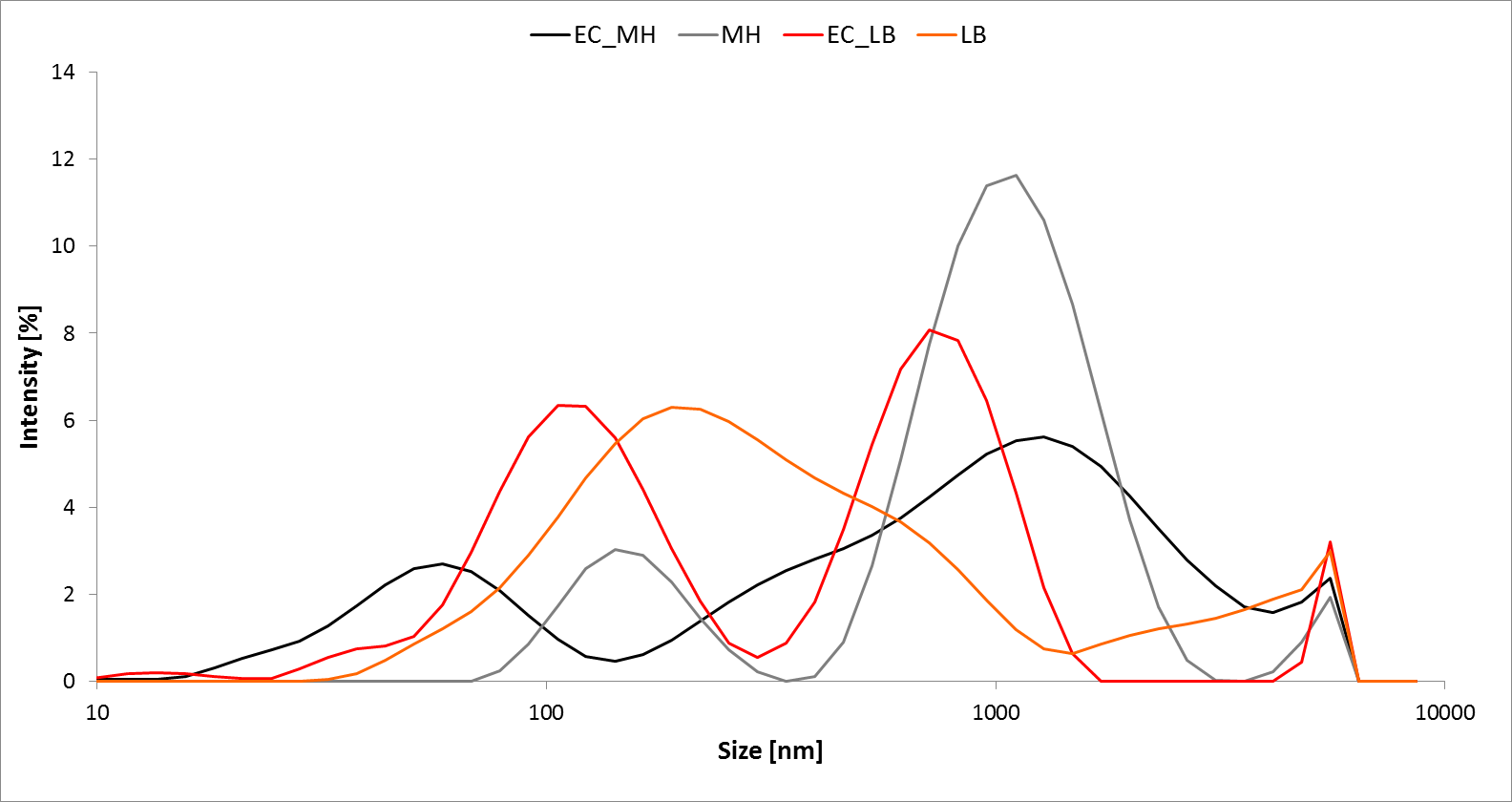


**Figure S3a.** Size distributions of LB and MH broths compared to *E. coli* liquids in these media


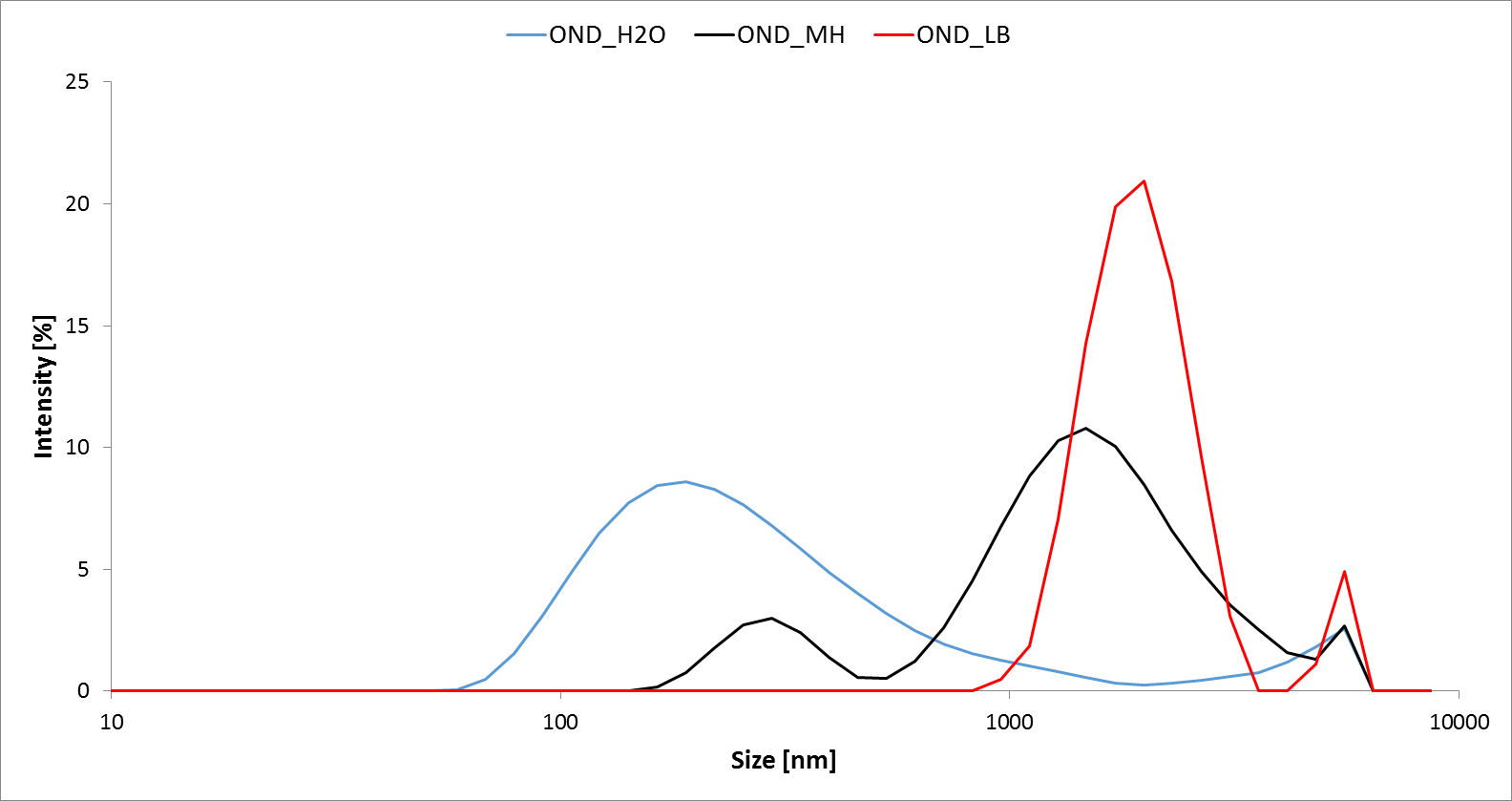


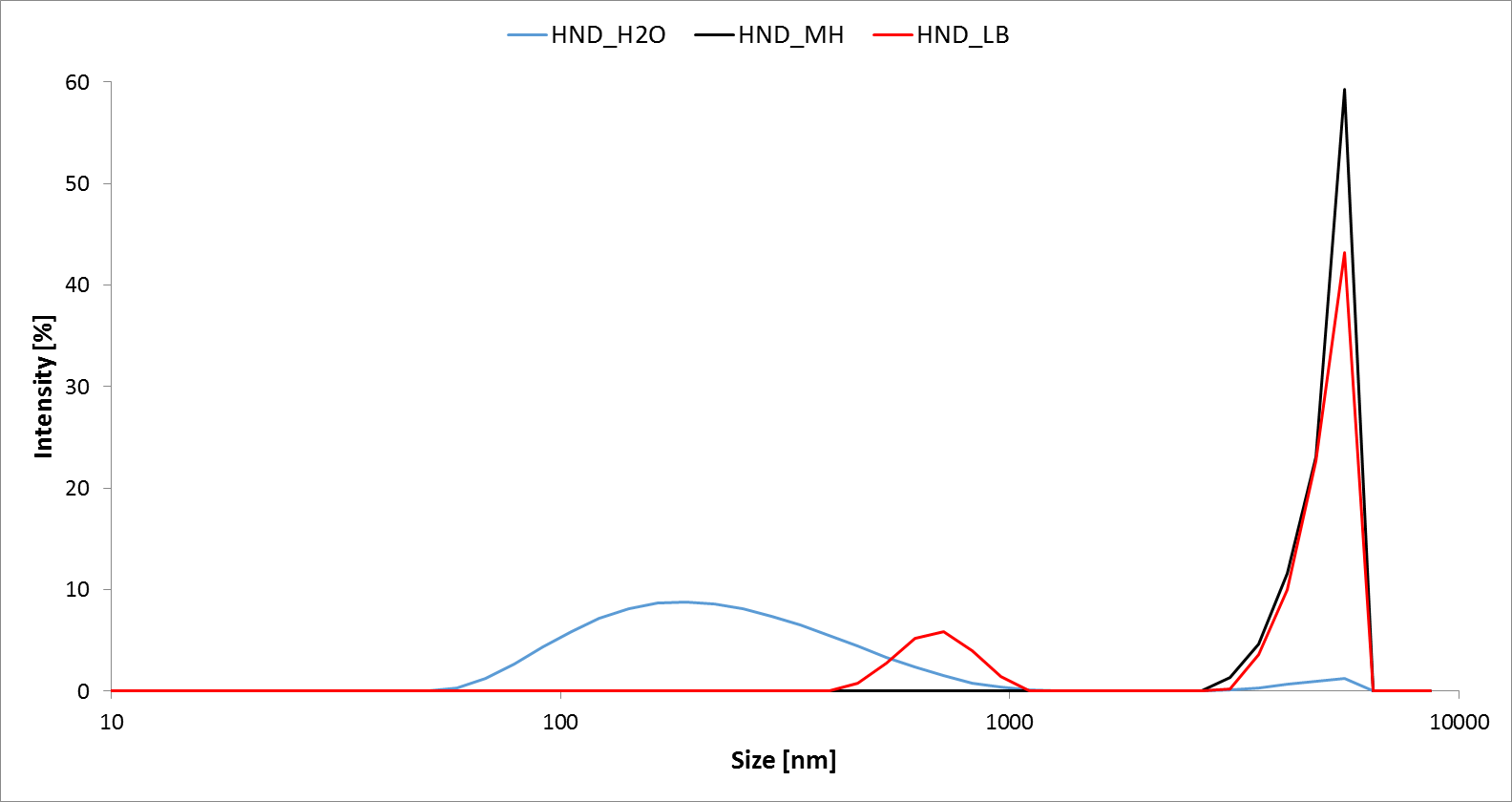


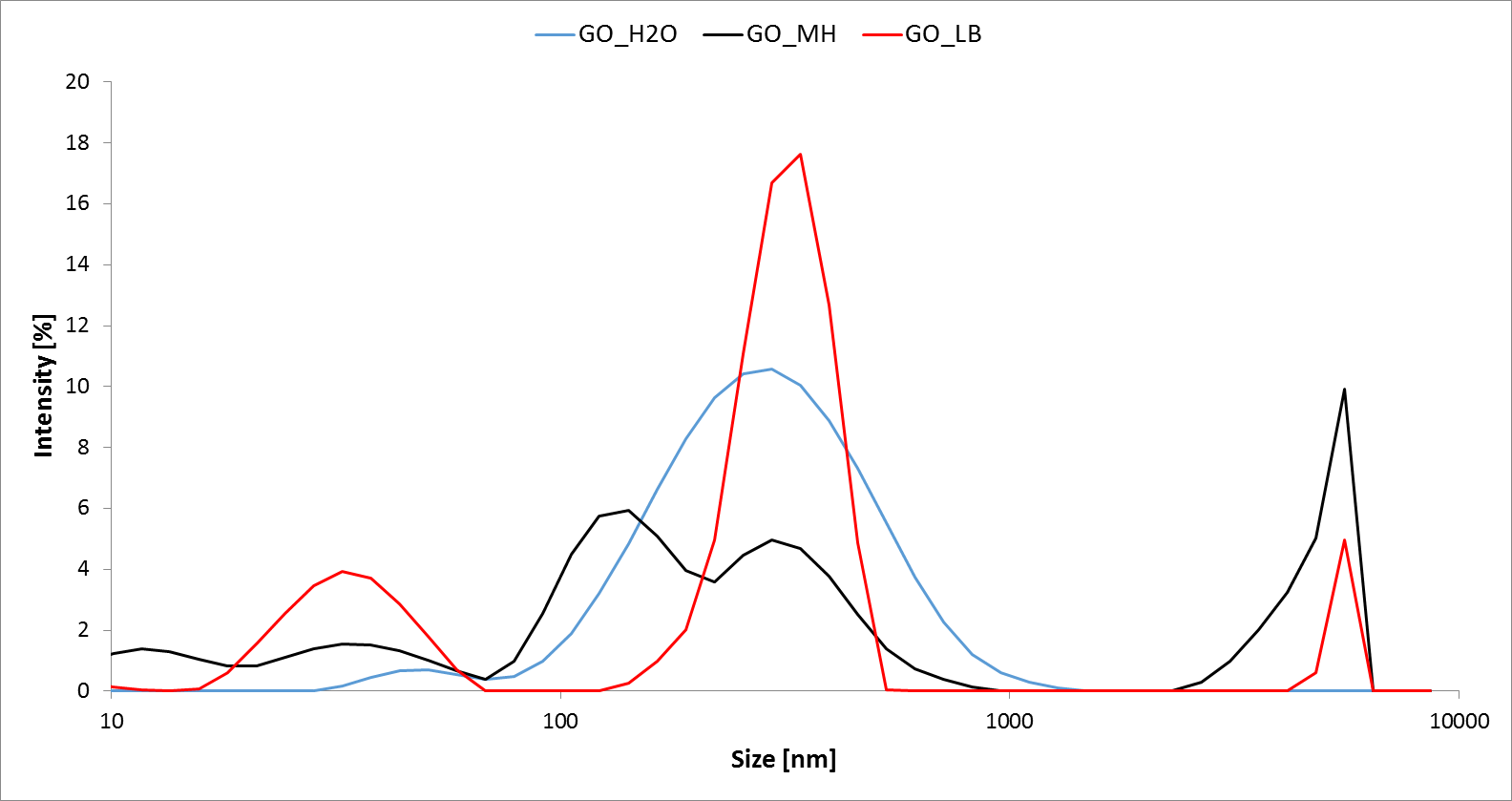


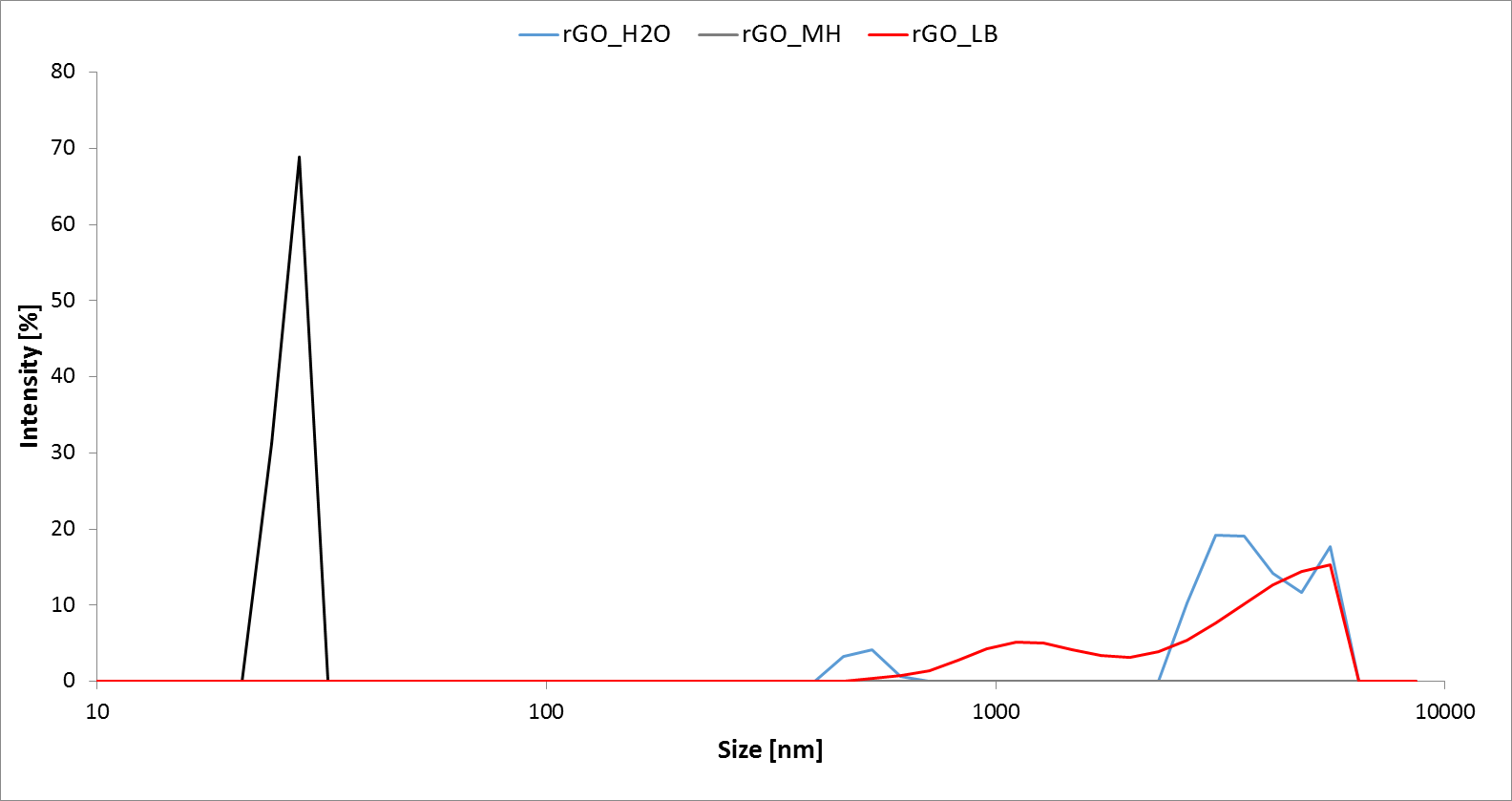


**Figure S3b.** Size distributions of nanomaterials in water and LB, MH broths
